# Biophysics and Clinical Effectiveness of Irreversible Electroporation for Catheter Ablation of Atrial Fibrillation

**DOI:** 10.3390/jcdd12060218

**Published:** 2025-06-07

**Authors:** Anna-Sophie Eberl, Gernot Plank, Martin Manninger, Ursula Rohrer, Laura Stix, Stefan Kurath-Koller, Andreas Zirlik, Daniel Scherr

**Affiliations:** 1 Division of Cardiology, Medical University Graz, 8036 Graz, Austria; martin.manninger@medunigraz.at (M.M.); u.rohrer@medunigraz.at (U.R.); laura.stix@uniklinikum.kages.at (L.S.); andreas.zirlik@medunigraz.at (A.Z.); daniel.scherr@medunigraz.at (D.S.); 2 Department of Physics and Biophysics, Medical University Graz, 8036 Graz, Austria; gernot.plank@medunigraz.at; 3 Division of Pediatric Cardiology, Department of Pediatrics & Adolescence Medicine, Medical University Graz, 8036 Graz, Austria; stefan.kurath@medunigraz.at

**Keywords:** ablation, atrial fibrillation, biophysics, pulsed electric field, pulsed field ablation

## Abstract

Understanding the biophysics of electroporation—the mechanism by which pulsed electric fields achieve tissue ablation—is essential for advancing this emerging technology. In this review, we summarize key publications from past years to provide an overview of current knowledge and future perspectives. We discuss the fundamental principles of PFA at the cellular, physical, and technical levels, along with its potential benefits and limitations. A deeper understanding of these biophysical mechanisms and the parameters required to create durable lesions may contribute to improved clinical outcomes and drive future innovation.

## 1. Introduction

Atrial fibrillation (AF) is the most common sustained arrhythmia in adults, accounting for a substantial portion of overall cardiovascular costs. With the prevalence of AF expected to double over the next few decades, prioritizing AF care and expanding access to catheter ablation (CA) as a rhythm control strategy are essential to meet the growing demand [1]. It is known that early rhythm control enhances both symptom control and quality of life [2]. Furthermore, it has been shown that early rhythm control lowers the risk of adverse cardiovascular outcomes, especially in patients with heart failure [3,4]. CA as a rhythm control strategy was superior to pharmacological treatment in various studies [5,6]. Therefore, CA is the preferred strategy for rhythm control, particularly in patients with paroxysmal AF. In those with symptomatic persistent AF, CA should be the treatment of choice if antiarrhythmic medications fail [1,4,5]. As the number of patients with a clear indication for AF-CA is continuously increasing, the procedure should be accessible, efficient, safe, and effective.

Pulsed field ablation (PFA), as a promising method for AF ablation, has been used in daily clinical practice for at least five years. Its encouraging safety profile, shorter procedure times, and comparable effectiveness compared to thermal methods [7,8,9,10] have led to a significant increase in operators using PFA for rhythm control in their AF patients. The reproducibility of PFA and its relatively short learning curve have made it an appealing choice, prompting more centers to incorporate this method into their electrophysiology (EP) labs.

While significant progress has been made, it is crucial to understand the mechanisms and biophysics behind this technique to fully appreciate its benefits and limitations.

## 2. Methods

A systematic review based on current and relevant literature of clinical and preclinical studies evaluating the biophysics, efficacy, and effectiveness of PFA was carried out. To ensure inclusion of the most recent findings, a structured literature search was performed in PubMed using combinations of keywords “pulsed field ablation”, “electroporation”, “transmembrane voltage”, “tissue conductivity”, and “electric field distribution”. The search was restricted to articles published in peer-reviewed journals in English, and preference was given to studies published within the last 10 years. Reference lists of key articles were also screened to identify additional relevant publications. Both basic science studies and clinical investigations were included to provide a broad perspective on the topic. As randomized controlled data are rare, only one RCT could be included.

## 3. Biophysical Mechanisms of Pulsed Field Ablation

Reversible electroporation has been used for several decades, initially in the field of oncology to temporarily enhance the permeability of tumor cell membranes (CM), thereby facilitating the uptake of molecules (e.g., chemotherapeutics) into various tumor tissues [11]. Reversible electroporation is also used for gene electro-transfer (e.g., DNA vaccines and gene therapy) and transdermal drug delivery [12,13]. In 2015, Martin et al. demonstrated that irreversible electroporation, leading to cell death, is an effective method to prolong survival in patients with locally advanced pancreatic cancer [14].

Animal experiments investigating cardiac ablation by electroporation commenced in the early 1980s. The effect of intracardiac direct current shocks was investigated, discovering that these shocks caused the formation of nano-pores in the cell membranes of cardiomyocytes, leading to apoptosis [11]. Due to the significant complication profile at the time, including barotrauma and arching, its clinical use was discontinued [15]. Nevertheless, preclinical investigations went on, and over time, it became evident that using lower energy amounts and applying the electric field (EF) in multiple short-duration pulses (nanoseconds to microseconds) enabled irreversible electroporation (IRE) without the occurrence of the above-mentioned complications [13].

### 3.1. Basics of Cell Electrophysiology

**Figure 1:**
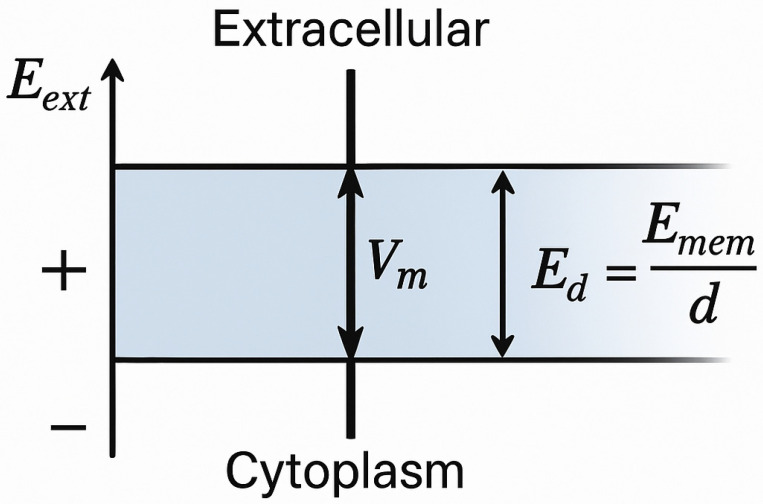
The cell membrane can amplify EFs applied across it, resulting in the generation of large transmembrane voltage. The CM acts as a dielectric film between two conductive media—the cytoplasm and extracellular space. The applied external EF (E_ext_) induces a redistribution of charges, resulting in the buildup of a transmembrane voltage (V_m_). The stronger the external EF (E_ext_), the greater the transmembrane voltage (V_m_)—and consequently, the higher the likelihood of electroporation. Since the membrane is extremely thin, even a small V_m_ leads to a high EF strength within the membrane (E_d_), according to E_d_ = V_m_/d.

The CM is composed of a bilayer of phospholipids that surround the cytoplasm as a dielectric border. The hydrophilic heads of these phospholipids are oriented towards the internal and external surfaces of the cell, allowing water to diffuse, while charged ions and larger molecules are impermeable [13]. Due to its low conductivity, the CM amplifies the externally applied EF, resulting in a high transmembrane voltage that manifests as a strong local EF across the thin membrane (Figure 1).

**Figure 2:**
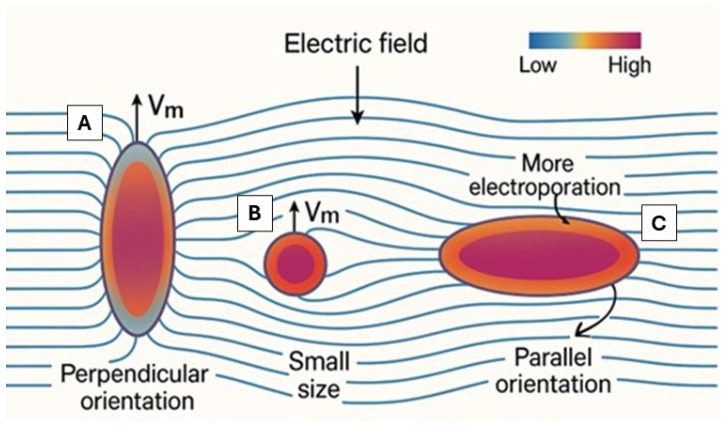
Different effects of the EF on different structures, depending on the orientation (**A**,**C**) and the size (**B**) of the structure. (**A**): A cell oriented perpendicularly to the EF has a strong field concentration at its poles. This leads to higher transmembrane voltage (V_m_) at these sites and makes the cell more susceptible to electroporation. (**B**): Small, spherical cells generally require a higher external field to reach the same V_m_ as larger ones. Although they respond more quickly, the magnitude of V_m_ is lower due to their reduced membrane surface area. (**C**): A cell oriented parallel to the EF experiences a more uniform distribution of V_m_ around its membrane. However, electroporation tends to occur more widely across its surface, particularly in areas where the field interacts tangentially [22].

Simultaneously, the charged phospholipid’s heads align with the EF’s vector, which can disrupt the CM’s architecture. This can lead to leakage, unregulated ion inflow and outflow, and ultimately cell death [16,17]. The impact of an EF on the CM can vary depending on the amplitude and direction of the vector, as well as on parameters such as cell geometry, orientation, and dimension [18]. Cells that are oriented perpendicular to the EF become damaged irreversibly at lower voltage than parallel cells [19]. Also, smaller cells require higher EFs to achieve the same transmembrane voltage as larger ones [20]. They tend to alter their transmembrane voltage more quickly but to a smaller extent [21]. It has also been observed that the cell’s survival is related to the proportion of the surface electroporated, highlighting the significant role of cell geometry and orientation in determining the outcome [20] (Figure 2).

### 3.2. Irreversible Electroporation

**Figure 3:**
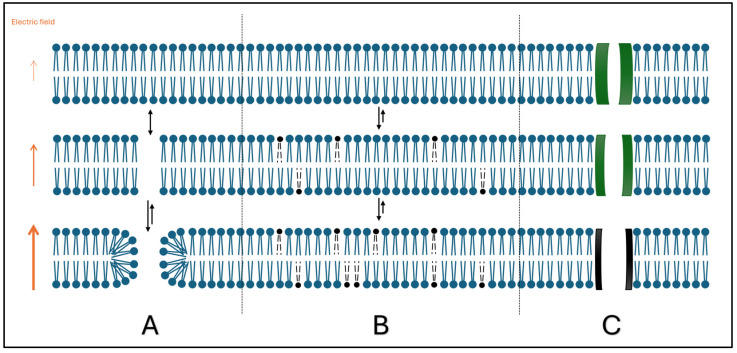
Mechanisms of pore formation at CMs when exposed to an EF (adapted from [25]): (**A**) reorientation of phospholipid head; (**B**) field-induced chemical changes of phospholipid tails; and (**C**) loss of ion channel functions.

When submitting electric energy via a lead or a catheter between at least two electrodes in contact with myocardial cells, a transmembrane voltage is induced, resulting in a shift of ions across the membrane. Depending on the strength of the EF and the total electrical resistance, which affects the change in transmembrane potential (related to the action potential), the cells may or may not become excited [23,24]. The stronger the field, the greater the transmembrane potential and therefore the greater the impact on and reaction of the cell, respectively. Applying voltage ranges that lead to a sufficient transmembrane potential change results in a fast depolarization due to an abrupt influx of sodium into the cells [23]. If a certain threshold is exceeded, irreversible nanoscale membrane defects or “pores” are created (electroporation), causing disruption of the cellular membrane integrity by changing its permeability [16,17,25]. When the CM is exposed to an external EF, the probability of pore formation increases, as the energy required for water molecules to penetrate the phospholipid bilayer decreases. This phenomenon is directly proportional to the external field strength to which the CM is exposed. It leads to reorientation of the lipophilic phospholipid heads towards the water molecules, forming metastable hydrophilic pores (Figure 3A). Moreover, exposure to an EF has been shown to induce chemical changes that result in the deformation of the lipid tails in the cell membrane, which consequently leads to an increase in permeability (Figure 3B). The application of high-voltage electrical pulses can also induce possible loss of function of pre-existent voltage-gated channels when exposed to an EF (Figure 3C) [25]. Altogether, this leads to the inability to preserve the cell’s homeostasis because of unregulated ion inflow and outflow [21]. Above all, disruption of calcium homeostasis and a significant increase in intracellular calcium seem to play an important role in IRE [19]. Various additional processes, such as change in tissue pH and mitochondrial cytochrome c release, are induced, thereby maintaining cell death [16,17,21]. Histologically, necrotic cardiomyocytes are replaced by an inflammatory infiltrate after 1–3 days. After 7–14 days, the fibroblast infiltration transforms into a fibrotic matrix of extracellular proteins. After 21 days, a fibrotic scar has developed, leaving no viable cardiomyocytes, while interstitial nerves and small blood vessels remain functional within the treated areas [21].

## 4. Field Strength and Tissue Effects

Local field strength—and consequently the irreversibility of electroporation, cell recovery, and safety—varies depending on several parameters, even when the applied voltage remains constant. The quality of the lesions, including their depth and transmurality, depends on the irreversibility of electroporation and the subsequent cell death. To achieve irreversibility and safety at the same time, various parameters are important, which will be discussed in the following paragraphs [21]. Varying these parameters alters the effect of the EF, depending on whether electroporation will be reversible or irreversible. Finally, depending on the strength of the resulting EF, thermal effects may occur [15,26]. It is important to remember that the effectiveness and safety of PFA can only be guaranteed if the parameters are appropriately adjusted to avoid thermal effects. Therefore, it is crucial to note that the optimal parameters are different for each PFA system. Furthermore, it should be emphasized that the response of different tissues or cell types to a given EF should never be compared without taking into account the PFA protocol used [21], as different tissues have specific thresholds, above which IRE induces necrosis and apoptosis (Table 1). Since cardiac cells appear to have the lowest threshold, cardiomyocytes seem to show relative sensitivity to PFA. Due to their lower energy threshold, adjacent vulnerable tissues, such as nerves, endothelial cells, red blood cells, and vascular smooth muscle, are less affected [26,27,28,29]. Myocardial tissue is primarily comprised of myocardial cells, but depending on the distinct myocardial disease, it may also contain varying amounts of fibroblasts, collagen, adipose tissue, endothelial cells, and inflammatory cells. As cell death by IRE at a certain voltage is selective to myocardial cells, the remaining tissues’ integrity seems to stay intact. This, together with the prevention of chronic atrial fibrotic changes and a lower inflammatory response compared to thermal ablation methods, may explain why tissue compliance is preserved and left atrial mechanical function may even improve after PFA [30,31].

### 4.1. Parameters That Define Electric Field Strength and Electroporation Effects

#### 4.1.1. Voltage

According to various studies, any EF strength between 400 V/cm and 1200 V/cm would be sufficient to trigger electroporation in myocardial cells while sparing adjacent tissue [32,33,34]. Safety has been demonstrated in preclinical studies up to an EF of 2000 V/cm [35]. An increase in the EF strength beyond this value would only lead to thermal effects according to Joule’s law: If a current (I) flows through a conductor with resistance (R) for time (t), the heat developed in the conductor is equal to the product of the square of the current, the resistance, and the time. In other words, the power of heating is directly proportional to the resistance, the square of the current, and the time the current is applied (Heat = I^2^ × R × t) [13]. Zang et al. demonstrated in a rabbit model that pulse amplitude significantly impacts temperature rise. However, the number of pulse trains and the number of pulses within a pulse train also significantly contribute to temperature rise [36].

Further increases in voltage than 1200 V/cm would negatively impact muscle contraction, alter the volume of microbubbles, and increase the likelihood of electrical arcing, which can lead to char and coagulation formation or barotrauma. Regarding muscle contractions, again voltage amplitude, pulse interval, and the number of pulses in a pulse train contribute significantly. However, the number of pulse trains does not appear to be a significant parameter [36]. The effect or strength of an EF therefore depends not only on the voltage applied but also on the time and the duration of exposure to a particular EF. Energy can be delivered either monopolarly, where energy is delivered between each electrode and the back patch, or bipolarly, wherein energy is delivered between the catheters’ electrodes. In the latter, the energy is shifted so that the effects become additive. Research is currently being conducted into the combination of unipolar and bipolar energy delivery.

#### 4.1.2. Waveform Shape

**Figure 4:**
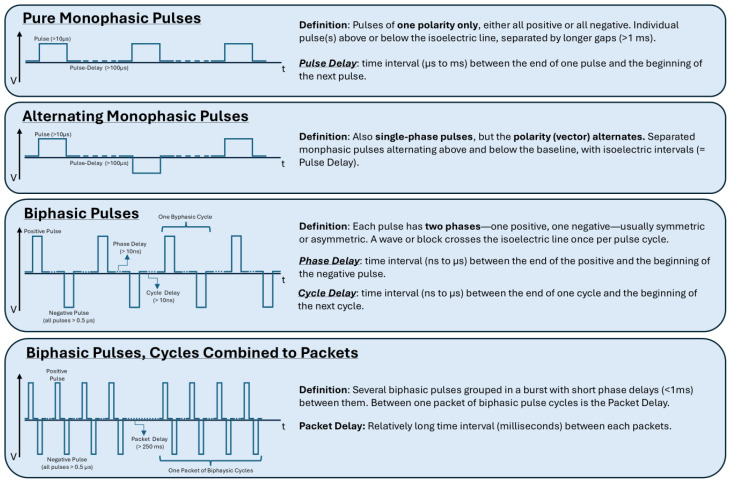
Visualization of different PFA waveforms.

Generating an EF requires rapid charging and discharging of the electrical energy stored in the capacitor (Figure 4). The very first PFA generators were designed to deliver monophasic pulses. Purely monophasic means a sequence of short (≤10 μs) or long (>10 μs) pulses delivered with relatively long (>1 ms) delays between them, all of which have the same polarity. Alternating monophasic pulses are a sequence of long pulses with relatively long delays between each pulse and alternating vectors, but again a pulse consists of one phase [21,37]. Lavee et al. were the first to perform and publish research on PFA for surgical epicardial atrial ablation in 2007. They used a monophasic direct current pulse sequence of 1500–2000 V pulse strength and a pulse duration of 100 μs per pulse, leading to transmural lesions [38]. However, dangerous adverse events have been reported using monophasic pulses. Monopolar waveforms have the strongest treatment effect because they result in the greatest charging capacity but can potentially cause muscle contraction, possibly leading to electrode displacement, as well as pressure waves and gaseous emboli formation by electrochemical reactions on the electrode surface [21,39]. Procedures using monophasic waveforms often require general anesthesia and paralytic agents because of extensive muscle contraction. In the analysis of two of the earliest clinical PFA trials published in 2019, Reddy et al. reported that all patients in whom monophasic waveforms were used during ablation, general anesthesia, and neuromuscular paralysis were necessary [37].

Biphasic current means that short (≤10 μs) pulses with relatively short delays (<1 ms) are combined with changes in polarity to form a cycle. Biphasic waveforms show less muscle contraction but provide a weaker treatment effect for an equivalent total packet duration and applied field [21]. Trials that investigated optimized waveforms for PFA deliveries could show that less patient movement and more durable pulmonary vein isolation (PVI) rates after three months could be achieved with an optimized biphasic waveform [37].

As the canceling effect of biphasic pulses requires high pulse amplitudes to be effective compared to monopolar pulses, scientists designed asymmetric waveforms. These waveforms appear to be more efficient in terms of depth and energy delivery, as they consist of positive and negative pulses with different pulse widths, which positively influences the cancelation effect. This results in lower energy requirements and thus the same treatment effect, but with a lower risk of side effects [39,40].

#### 4.1.3. Pulse Parameters and Duration

In summary, the EF strength depends on pulse intensity and waveform, which together cause cell dysfunction through the application of progressive pulse trains. Simplified, the effects are determined by the strength of the EF and the duration of exposure to this EF [21]. By delivering electric energy in pulses of short duration (microseconds to nanoseconds), heating issues can be minimized or potentially avoided [41]. Multiple pulses, applied over a time period of nanoseconds to milliseconds, can be summed up to a packet of pulses. Both the cumulative energy and treatment effect depend on the number of delivered pulses and the duration of the packet of pulses, but also on the number of subsequent packets delivered (Figure 4). However, Zang et al. could demonstrate that, besides pulse amplitude, the number of pulse trains and the number of pulses in a pulse train significantly contribute to all ablation outcomes [36]. These parameters also affect the chance of muscle contraction, heating, or gas formation. Pauses with a duration of milliseconds to tens of seconds between delivered packets allow the tissue to decrease temperature before the next delivery, preventing cellular recovery from affecting the lesion’s geometry in a negative way [21].

These secondary pulse parameters, which relate to the shape, sequence, and temporal structure of the pulses, are important to properly adjust the EF in order to increase efficiency and avoid thermal effects, as these electrical pulse delivery settings also determine the EF [13,18]. Only recently could a linear relationship between voltage and lesion depth, as well as number of cycles and lesion depth, be shown in a swine model [42]. However, waveform and pulse parameters must be chosen to deliver energy below a threshold that will cause cell death by thermal damage.

### 4.2. Catheter and Electrodes

Efficiency, and therefore lesion durability, also depends on catheter design, electrode size, and inter-electrode spacing. Catheter design, that is, the arrangement of the electrodes and the way they are coupled (bipolar between adjacent electrodes, electrode against remote diffusive patch), determines the geometry of the EF, and this EF is linked to changes in transmembrane voltage by the activating function [43,44].

In addition, the required contact force (CF) can vary between different catheter designs. The total electrical resistance is affected by the size, shape, and surface structure of the electrodes, as well as by inter-electrode spacing and any inter-electrode structure or tissue and their resistive properties that contribute to electrical conductivity [24]. Pulse design must be adapted and studied for each catheter design.

### 4.3. Resistance and the Impact of Tissue Contact and Contact Force

Unlike radiofrequency (RF) ablation, the effectiveness of PFA is less dependent on tissue impedance [15] and therefore appears to be more effective in scar tissue [45]. RF catheters require a certain level of tissue resistance to effectively heat tissue. Lower CF or lower impedance tissue, such as scar or fibrosis, will prevent effective energy delivery [46]. Younis et al. demonstrated that in a swine model of healed myocardial infarction, PFA applications produced more uniform, larger, deeper, and therefore more transmural lesions than RF applications [45]. This makes PFA suitable for creating contiguous lesions even in challenging areas, like scar tissue and heavily trabeculated areas in the ventricles or papillary muscles [15,47]. In a swine model, Nies et al. demonstrated that monopolar PFA with a lattice tip catheter reliably creates lesions on intracavitary structures, including the papillary muscles and moderator band, without safety issues in swine. They demonstrated that PFA could create deep, wide lesions at the epicardium and facilitate transmural left ventricular lesions with tip-to-tip bipolar catheter ablation [47].

On the other hand, there is evidence that adipose interference prevents effective lesion creation [42]. These findings become even more important when PFA lesions are aiming at ventricular or epicardial areas and need further investigation.

However, it has been shown that lesion depth and durability are directly proportional to catheter–tissue contact. Therefore, achieving good catheter-to-tissue contact is crucial, as lesions tend to become deeper with better contact [48,49]. Meckes et al. were able to show that the effect of electroporation diminishes with increasing distance of the catheter from the myocardial surface. This finding was consistent even when the voltage was increased [11]. These results could also be reproduced by Gasperetti et al., showing that contact was a key component for lesion formation. Lesions were significantly attenuated only at a catheter-to-tissue distance of 0.5 mm, while no lesions were observed at a distance of 1 mm. Again, the use of higher voltage protocols at the given distances did not lead to an improvement in lesion quality [42]. Importantly, these findings are independent of the catheter design. At high CF (15–30 g), representing full contact, lesion quality improves, with lesion depths of up to 5 mm [50]. These results are supported by Nakagawa and colleagues, who were the first to demonstrate that higher CF results in significantly deeper lesions in histochemical examinations of acute PFA lesions in swine ventricles. Interestingly, they observed no significant relationship between impedance drop and lesion depth, which would be a typical finding in RF ablation [51]. In summary, proximity to tissue is important. However, it is unclear whether CF itself makes a difference when you are in close proximity.

A decrease in tissue impedance is observed during the application of a pulse as the tissue becomes increasingly electroporated. Initially, cell membranes act as electrical insulators, implying a high impedance, resulting in a drop in impedance as the CM becomes permeabilized, the overall conductivity increases, and therefore the impedance decreases [52,53].

To date, no system provides accurate information on catheter–tissue contact, which represents a crucial area for improvement in the design of future catheters. Therefore, developing catheters with localized impedance tracking to assess tissue contact appears to be a high priority. Currently, operators help themselves by checking the alignment of the catheter to the vein alignment using fluoroscopy, intracardiac ultrasound, or by implementing mapping systems to incorporate local electrograms as a surrogate for tissue contact, as well as with fine motor tactile sense, which provides perceptible feedback about tissue contact. In some catheters, tissue contact is already provided by evaluating impedance changes between certain electrodes and the indifferent electrode [54]. ECLIPSE-AF evaluated lesion quality using standard contact force-sensing solid-tip ablation catheters with an open platform PFA generator. The authors report progressive improvement in PVI after 3 months of remapping and 1 year of clinical follow-up in those patients treated with the optimized PFA workflow and where digital information in CF was provided [55].

### 4.4. Repetition Dependence

Multiple clinical and preclinical investigations have focused on prediction of lesion formation and quality. Recently, the dependence of lesion creation on pose, contact, and repetition with a pentaspline catheter was confirmed in a swine model [56]. In both vegetable and swine models, an increase in lesion depth was observed when repeated applications were performed at the same catheter position [50,57]. Yavin et al. also demonstrated that PFA lesions in ventricular myocardium significantly expanded in lesion volume and depth if repetitive applications were performed at a similar EF in a porcine model. Histologically, lesions showed replacement of cardiomyocytes by collagen fibers and fat cells without interspaced myocardium [58,59]. Coagulative necrosis, as it would be the case in RF, was not observed [59]. Furthermore, in-human trials could also reveal the effectiveness of repetitive PEF applications. For example, the PULSE-EU study demonstrated a significant increase in durably isolated pulmonary veins when three applications were provided instead of one [60].

### 4.5. Clinical Implications and Future Directions—Combined Energy Lesion

Various preclinical trials are focused on improving the efficacy of creating durable and transmural lesions while enhancing safety. Verma et al. published on combining PFA with ultra-low-temperature cryoablation in six swine. Biphasic, bipolar PFA trains were applied after pre-cooling the tissue for 30 s. This led to 6–7 mm deep and, in large parts, transmural lesions, with no further occurrence of muscle contractions and microbubbles [61]. Researchers are also exploring combinations of RF and PEF energy to improve lesion quality. Lesions become significantly wider and deeper when combining both energy sources [62].

## 5. Safety Profile

Notably, PFA minimizes damage to surrounding structures like nerves, the esophagus, and blood vessels due to its tissue selectivity. When used cautiously, it also prevents thermal injury. Daniels and Rubinsky published on nerves’ resilience to PEF. They could show that the fatty, low-conductive myelin sheath seems to protect nerve cells by absorbing most of the voltage drop. Furthermore, axons are able to remyelinate via Schwann cells, which means that even if the myelin sheath becomes damaged, recovery of the axons is possible [63]. Several clinical and preclinical studies also showed sparing of esophageal tissue. A systematic review by Nies et al. showed that most studies in which supratherapeutic PEF was delivered from either the inferior vena cava, the right atrium, or the aorta to the esophagus or any cardiac chamber reported no esophageal damage. Two studies found damage to the esophagus, but examination was performed only at 2 and 48 h after the application, without long-term follow-up to see if these findings developed into chronic damage or resolved. Notably, different PFA systems were used in these two studies, and lesions could not be reproduced in surviving animals. However, in three trials where PEF was directly applied to the esophagus, the authors found acute small lesions that were described as inflammatory reaction, superficial edema, and focal necrosis. In those animals that received follow-up, lesions completely resolved after 7 days. Bigger acute lesions that were seen in rabbits after application of a strong EF (monopolar PFA; 70 μs pulse duration, 9 trains with 10 pulses each, 2.5 kV/cm) also did not show any long-term or sustained esophageal damage [64]. Kirstein et al. published on intraluminal esophageal temperature changes during PFA with a pentaspline catheter. The medical temperature increase was 0.8 ± 0.6 °C. The largest change was 3.7 degrees; the highest intraluminal temperature measured was 40.3 °C. All patients remained asymptomatic; none showed clinical signs of esophageal injury [65]. In conclusion, atrio-esophageal fistula seems to be unlikely but not impossible. There is also growing evidence that PFA does not lead to pulmonary vein (PV) stenosis. Several retrospective registries, but also randomized controlled studies, could reveal the absence of PV narrowing after PFA [7,9,15,66].

### 5.1. Possible Complications Associated with Pulsed Field Ablation

A recently published study comparing reported complications during PFA and RF ablation showed that the most common adverse events in PFA procedures are pericardial effusion (with 88.7% requiring pericardiocentesis or surgical drainage), vasovagal response, and hemolysis. However, pericardial effusion was not uncommon but occurred in less than half the cases compared to RF ablation [67]. A retrospective observational study analyzing 17,000 PFA procedures in 108 centers showed results of 0.36% of pericardial tamponade requiring intervention and only 11.1% of them with the need for surgical treatment. In another 0.33% of procedures, pericardial effusion without the requirement of any intervention was reported [68]. Both reports also provide data of occurring vasovagal response manifesting with either atrioventricular block or sinus arrest/atrial asystole, which resolved in all cases, demonstrating that autonomic changes after PFA seem to be transient [67,69,70]. Various studies also report on significant intravascular hemolysis, hemoglobinuria, and possible, but rarely occurring, concomitant renal failure after PFA [8,67,68]. Recently published trials could show that the number of PFA applications and also post-ablation hydration are independent predictors for post-procedural renal impairment [8]. Popa et al. reported increasing hemolysis severity if the sum of PFA applications exceeds a number of 54 [71]. Nies et al. provided data that show low tissue contact to be a predictor of hemolysis, reflecting the extended exposure of the EF to the blood pool [72]. These results could lead to the suggestion that hemolysis may be less likely when using monopolar PFA systems, since the energy is delivered directly to an indifferent electrode, thereby minimizing the EF’s exposure to the blood pool. However, data directly comparing the effects of bipolar and monopolar systems on hemolysis are currently lacking.

Another concern is the repeatedly described complication of coronary artery spasm when ablating in the immediate vicinity of these arteries [68,73,74,75,76]. Spasms occur due to electrically induced contraction of the vascular smooth muscle located within the tunica media of arterial blood vessels. As a result of various alterations to intracellular calcium and disruption of active membrane processes that regulate calcium levels, these spasms tend to be long-lasting. The occurrence of spasm depends on the proximity of the catheter to the vessels. To create IRE, the applied EF around the electrodes is substantially higher than the minimum IRE threshold for cardiomyocytes [77]. Pretreatment with nitroglycerin, either intravenous or intracoronary, may prevent spasms in some cases, but case reports of occurring spasms led the manufacturers to recommend avoiding ablation in regions such as the cavo-tricuspid isthmus [74,76]. It has been shown that adenosine triphosphate blockers and adrenoreceptor antagonists are ineffective at preventing coronary artery spasms [77].

Only recently, safety concerns have arisen from observations of coronary artery stenosis with neointimal hyperplasia and tunica media fibrosis, which could be seen at optical coherence tomography (OCT) in 87.5% of vessels when PFA was directly applied [78].

### 5.2. Microembolism and the Fear of (Silent) Brain Lesions

Delivery of PEF in the bloodstream, which is an ionic solution, leads to the separation of water molecules. This results in hydrogen gas at the active cathode and chlorine gas at the active anode. Reduction in bubble formation is achieved by alternating PEF polarity during energy delivery. Gas can also result from vaporization. This occurs when the electric current causes a temperature rise in the solution through heating in accordance with Joule’s law, and the temperature exceeds the phase change value. Despite significant efforts to minimize gas formation, it remains a standard collateral effect and potential patient risk. The first randomized controlled trial comparing PFA to thermal methods provided low numbers of silent cerebral events, but higher total numbers in the PFA (3/34) than in the thermal (RF: 0/20; Cryo: 0/17; total: 0/37) group. In the PFA group there was one transient ischemic attack reported, and in the thermal arm one stroke occurred [79]. In a first-in-human study assessing a focal 9 mm lattice tip catheter, asymptomatic brain MRI screening was performed. This study revealed silent cerebral events (DWI-positive/FLAIR-negative) in 7.9% of cases and silent cerebral lesions (DWI-positive/FLAIR-positive) in 6.7% of cases [80]. Good tissue contact reduces the possibility of bubble formation, as less energy is applied to the bloodstream. Further investigations are required to determine whether modifications should be made to the current praxis.

## 6. Further Benefits/Advantages

PFA procedures seem to have a positive impact on quality of life directly after the procedure, with reported reductions in chest pain and a lower incidence of pericarditis, respectively, compared to thermal energies, in which these symptoms may occur. The incidence of pericarditis after RF or cryoballoon ablation for AF is described in 3.7–10.6% of patients [81,82,83]. In the MANIFEST-PF registry, evaluating 17,000 patients following CA with PFA, only 0.17% of patients developed acute pericarditis [68]. This finding is supported by data from the EU-PORIA registry, showing an incidence of 0.16% [8].

In addition, PFA procedures provide significantly shorter procedure duration [7,37,66,73], which implicates shorter sedation duration/general anesthesia time, potentially contributing to a quicker recovery afterwards.

## 7. Limitations of PFA and Further Questions for Science

### 7.1. Effectiveness and How to Further Increase It

So far, only short-term follow-up of PFA compared to thermal methods is available. Its true efficacy and safety profile must be evaluated in the future, as evidence is built through data and data are only gathered over time. Especially as controlled randomized data need to be gathered, we are eagerly awaiting the results of the first randomized European trial comparing PFA to RF [84]. With the integration of various PFA-enabled catheters into 3D mapping systems, ablation workflow could become even more efficient and precise. In addition, the pursuit of the possibility to provide information on CF could also help to improve the outcome after AF-CA using PFA.

Ablation of extracardiac tissue, such as performing epicardial cardioneuroablation, might be a promising future perspective, but there is not enough evidence yet [85].

### 7.2. Determining PFA Parameters for Prediction of Durable Lesions

Another open question is how and when to analyze successful isolation of PVs or lines during the procedure, as electrical stunning can affect the assessment for bidirectional block. Therefore, various clinical investigations on the prediction of lesion quality are ongoing. Duytschaever et al. observed residual sharp downward deflection in unipolar signals, consistent with the timing of the corresponding bipolar signal after PFA. This could be useful for predicting tissue response, as 30% of these signals diminished after further PFA applications. They conclude that persisting deflections could be a measure of non-isolation or reversible electroporation [86]. Data from Barcelona provide other insights on unipolar electrogram morphology and change after PFA. They suggest that especially the different recovery dynamics could serve as possible markers to predict reversible and irreversible electroporation [87]. A recently published paper showed again that unipolar electrograms (EGMs) can serve as guidance for transmurality or nontransmurality of PFA lesions by interpreting changes in bipolar and unipolar EGMs as well as in current injury. Interestingly, prediction by low-frequency (1–16 Hz) change, which was observed in transmural PFA lesions, was not seen in transmural RF lesions [88].

Another approach to lesion durability prediction was described by Martins et al. They used a novel unipolar PFA system and guided their ablation by optical imaging using polarization-sensitive optical coherence reflectometry (PS-OCR). To predict the quality of the lesion, they analyzed reflective characteristics of myocardial tissue and visualized the real-time contrast between healthy and ablated tissue. They interpreted the decrease in tissue birefringence, which depends on the different refractive indices of a medium due to different propagation directions and polarization of light. A drop of more than 17% predicted durable lesions during 3-month remapping studies [89].

Lastly, PV reconnections after PFA remain a significant issue. A recently published retrospective analysis of redo procedures showed that in 427 redo procedures, only 45% of patients had durably isolated PVs [90]. As PV isolation remains the cornerstone of AF catheter ablation, it is crucial to decrease the chance of PV reconnections in PEF ablation.

### 7.3. PFA for Ventricular Ablations

There is growing interest in using PFA for ventricular ablations. While no catheter is currently approved for clinical use in ventricular myocardium, various preclinical studies have demonstrated different lesion qualities and also frequent transmurality of lesions [21,45,47,51,56,58]. As PFA appears to create satisfying lesions even in scar tissue [45], it could be promising for future ventricular ablation. However, this requires further investigation, as the optimal pulse parameters and electrode design for ablating ventricular myocardium are yet to be determined.

## 8. Conclusions

PFA has already become part of routine clinical practice, supported by growing experience, ongoing research, and the continued evolution of PFA systems—highlighting a promising future for this technology. However, much remains to be understood. A deeper insight into the biophysics of PFA and the parameters required to achieve durable lesions will be essential for optimizing clinical outcomes and driving future innovation.

## Figures and Tables

**Table 1:** Different EF thresholds for IRE in different tissues allow ablation of specific tissues (adapted from [28]).

Tissue	Electric Field Threshold for IRE (V/cm)
Nerve	3800
Vascular smooth muscle	1750
Red blood cells	1600
Liver	700
Kidney	600
Pancreas	500
Myocardium	400

## Data Availability

No new data were created or analyzed in this study.

## Abbreviations

The following abbreviations are used in this manuscript:

AF	atrial fibrillation
CA	catheter ablation
CF	contact force
CM	cell membrane
EF	electric field
EGM	electrogram
I	current
IRE	irreversible electroporation
OCT	optical coherence tomography
PEF	pulsed electric field
PFA	pulsed field ablation
PS-OCR	polarization-sensitive optical coherence reflectometry
PV	pulmonary vein
R	resistance
RE	reversible electroporation
RF	radiofrequency
T	time
V	voltage
